# Marked variability in published CMR criteria for left ventricular basal slice selection - impact of methodological discrepancies on LV mass quantification

**DOI:** 10.1186/1532-429X-15-S1-P101

**Published:** 2013-01-30

**Authors:** Jamie Mullally, Parag Goyal, Lauren A Simprini, Anika Afroz, Jonathan D Kochav, Noel Codella, Richard B Devereux, Jonathan W Weinsaft

**Affiliations:** 1 Medicine/Cardiology, Weill Cornell Medical College, New York, NY, USA; 2 Medicine/Cardiology, Memorial Sloan Kettering Cancer Center, New York, NY, USA; 3 Duke University School of Medicine, Durham, NC, USA; 4 IBM Thomas J. Watson Research Center, Yorktown, NY, USA

## Background

Left ventricular mass (LVM) quantification by cine-CMR is typically performed by planimetry of contiguous LV short axis images. This approach relies on use of anatomic landmarks or quantitative binary cutoffs to define the basal-most aspect of the LV. Methodological discordance concerning criteria for LV basal slice selection has the potential to alter cine-CMR quantified LVM. This study assessed frequency of methodological variability in published LV basal slice definitions, as well as its impact on cine-CMR quantification of LVM.

## Methods

A Pubmed search was performed using the terms "left ventricular mass", "left ventricular hypertrophy", "myocardial mass", or "hypertrophy" AND "cardiac MRI", "CMR" or "magnetic resonance imaging" [species: human, language: English, publication date: after 1/1/00]. Manuscripts were reviewed by an AHA/ACC level III CMR trained physician for methodology concerning basal slice criteria; methods were categorized based on quantitative and anatomic criteria as defined in each manuscript. Published methods were then applied to an established registry of CAD patients: Cine-CMR images were planimetered for percent circumferential LV myocardium and basal-most LV short axis slices were assigned using the most common quantitative cutoffs determined by literature review.

## Results

**Figure 1:**
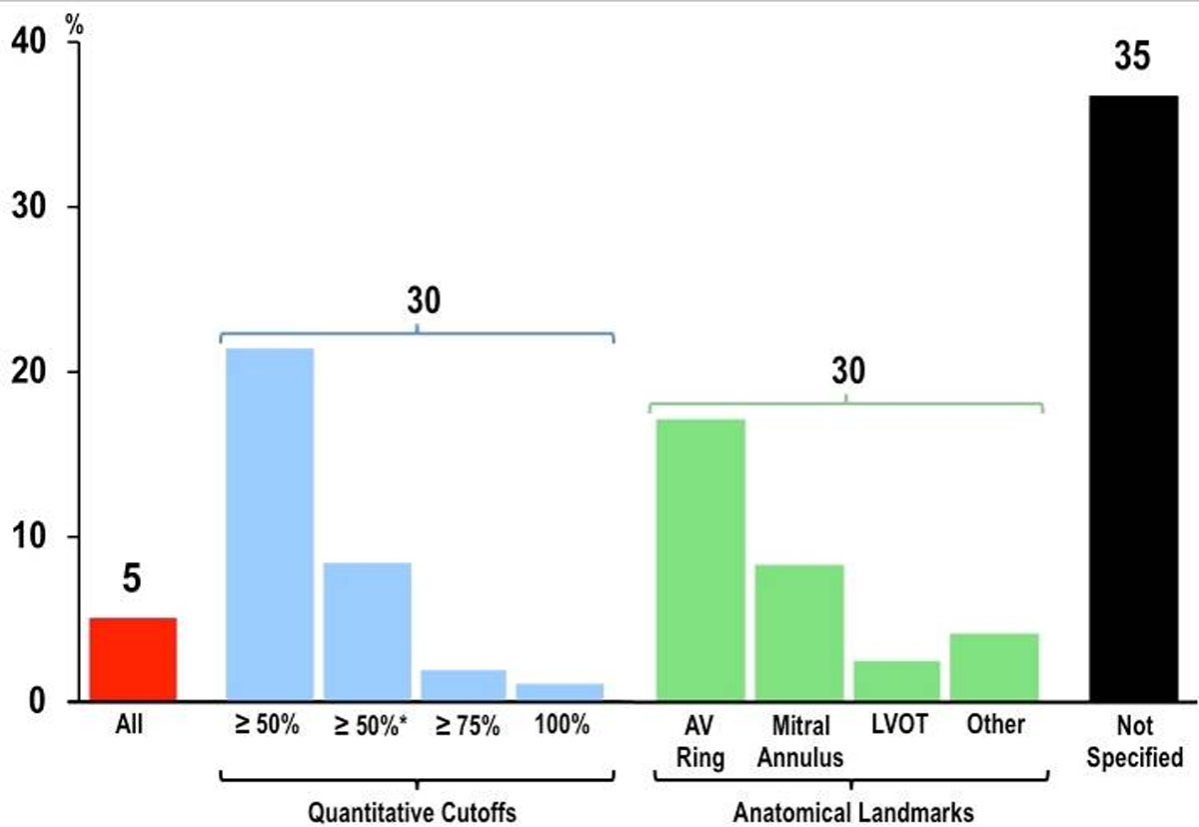
Frequency of different quantitative (blue bars) and anatomic (green) criteria for LV basal slice selection in published CMR literature. Note that basal slice criteria were frequently unspecified (black), and rarely included all LV myocardium (red) imaged on short axis SSFP.

**Figure 2:**
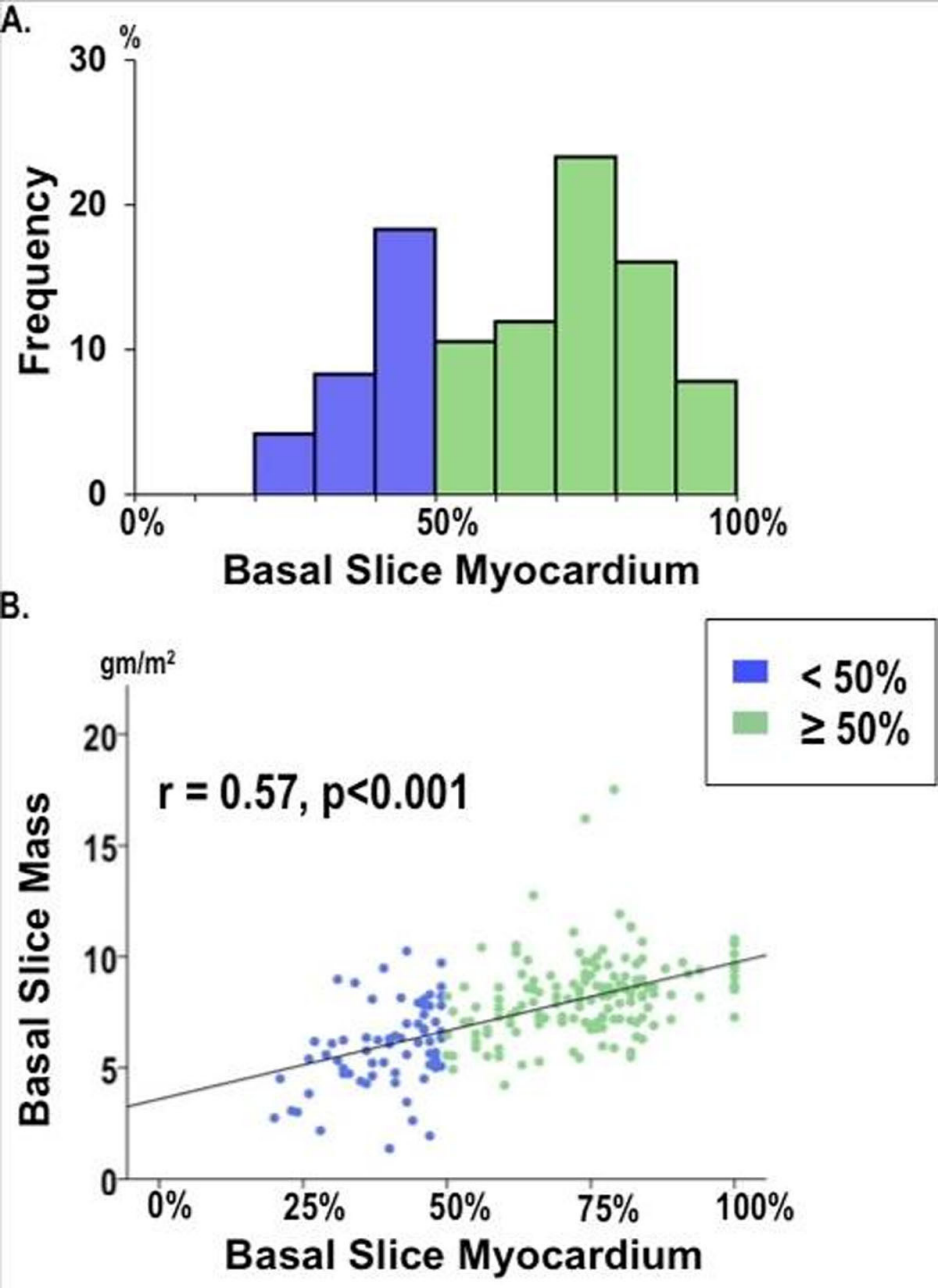
A) Distribution of circumferential extent of LV myocardium among patient cohort (blue <50%, green ≥50%). B) Scatter-plot demonstrating correlation between circumferential extent of LV myocardium and LV mass comprised within each basal short axis slice.

129 original research publications that measured LVM by cine-CMR were identified by systematic literature review. Basal slice criteria were unspecified in 35%, included all LV myocardium in a small minority (5%), with the remainder evenly divided in use of anatomic landmarks (30%) or quantitative cutoffs (30%) (Figure 1). Marked variability was present in both published anatomic and quantitative criteria for basal slice definition, with the latter most often defined using a binary cutoff of 50% myocardial circumference during end diastole alone (ED50; 20%), or during both end-diastole and end-systole (EDS50; 8%). Among 150 CAD patients (57±12yo, 83% male, 43% HTN), circumferential extent of basal slice LV myocardium varied: 31% of basal slices analyzed contained LV myocardium comprising <50% chamber circumference (Figure 2A). LVM contained within basal LV slices strongly correlated with circumferential extent of LV myocardium (r=0.57, p<0.001) (Figure 2B). LVM excluded using binary cutoffs constituted 5.4±6.5gm (2.7±3.2 gm/m^2^) for ED50 and 22.0±10.0gm (11.2±5.0 gm/m^2^) for EDS50, respectively constituting 3.2% and 13.0% of total LVM as calculated when all CMR-evidenced LV myocardium was included.

## Conclusions

Marked variability exists in published CMR literature regarding anatomic and quantitative criteria to define the basal-most LV on short axis cine-CMR. Application of established binary cutoffs frequently excludes LV myocardium from CMR measurements, resulting in 3-13% decrements in calculated LVM.

## Funding

K23 HL 102249-01, Lantheus Medical Imaging (unrestricted research grant)

